# Successful Treatment of Cholecysto-Antral Fistula: A Case Report

**DOI:** 10.7759/cureus.33580

**Published:** 2023-01-10

**Authors:** Raheel Anis, Ihab Elmesallami, Aisha Khan, Nuaman A Danawar

**Affiliations:** 1 General Surgery, Security Forces Hospital Program - Dammam, Dammam, SAU; 2 Vascular Surgery, Security Forces Hospital Program - Dammam, Dammam, SAU; 3 Clinical Observer Surgery, Security Forces Hospital Program - Dammam, Dammam, SAU

**Keywords:** case report, ct abdomen, gallstone ileus, gallstones, bowel obstruction, fistula, cholecystoantral fistula

## Abstract

Cholecysto-antral fistula and gallstone ileus are rare complications of a common disease, gallbladder stone (GBS). This fistula is developed as a prolonged complication of cholelithiasis in which the gallbladder adheres to the adjacent antrum, and a stone erodes through the wall. Among the variety of cholecystoenteric fistulae, the cholecystoduodenal fistula occurs more commonly than the cholesysto-antral fistula.

In this scientific study, we present a 98-year-old male patient who came to ER with a complaint of abdominal pain, vomiting, and constipation for five days. He was vitally stable and had normal laboratory results. The plain abdominal X-ray showed dilated loops with excessive gases. His computed tomography (CT) abdomen with contrast showed small bowel obstruction secondary to an impacted gallstone at the distal jejunum, fistulous communication between the gall bladder and the antrum, and pneumobilia.

Our management included endoscopic retrieval of a single gallstone from the second part of the duodenum followed by open surgical enterolithotomy, partial cholecystectomy, and closing of the fistula. Despite our case sharing many aspects with the available literature, our case, to our knowledge, is the first case of ileus gallstone occurring in a 98-year-old patient. Cholecysto-antral fistula has not been widely published in the literature. The offending gallstone presented along with the radiological Mercedes Benz sign which does not present in all cases of GBS. Typically, the obstructing GBS stops at the terminal ileum, but in our case, it was dislodged in the distal jejunum with no previous biliary symptoms. Finally, we were able to remove another single GBS from the second part of the duodenum during the preoperative upper endoscopy. The clinical diagnosis may be missed due to the vague presentation of symptoms; hence imaging, especially of the CT abdomen is crucial in establishing the diagnosis, moreover, performing an upper endoscopy could have diagnostic and therapeutic benefits. In cases like this, the main surgical intervention should be to address the bowel obstruction, and cholecystectomy with fistula closure may be added if the patient’s condition is stable with minimal inflammation and adhesion.

## Introduction

Gastro-antral fistula and gallstone ileus are rare complications of gallbladder stone (GBS) disease, which can lead to small bowel obstruction (SBO) secondary to the impaction of the GBS in the small bowel, typically in the terminal ileum. Usually, a large stone erodes through the gallbladder into the contiguous bowel, most frequently the duodenum, creating a cholecysto-enteric fistula (CEF) [1]. Through this CEF, the GBS passes distally and is dislodged in a narrowed area. The CEF manifests with broad-spectrum and nonspecific symptoms; hence, an accurate preoperative diagnosis may not be reached in all cases [2]. The CEF occurs at the average age of 70 years; the youngest reported age is 13 years and women are 3-16 times more likely to be affected [3]. Both rarity and atypical presentation make the diagnosis challenging and lead to delayed management and poor outcomes, especially in the absence of previous biliary symptoms. The main aim of this report is to increase the awareness of emergency department physicians, radiologists, and surgeons about this rare presentation of GBS.

## Case presentation

**Figure 1:**
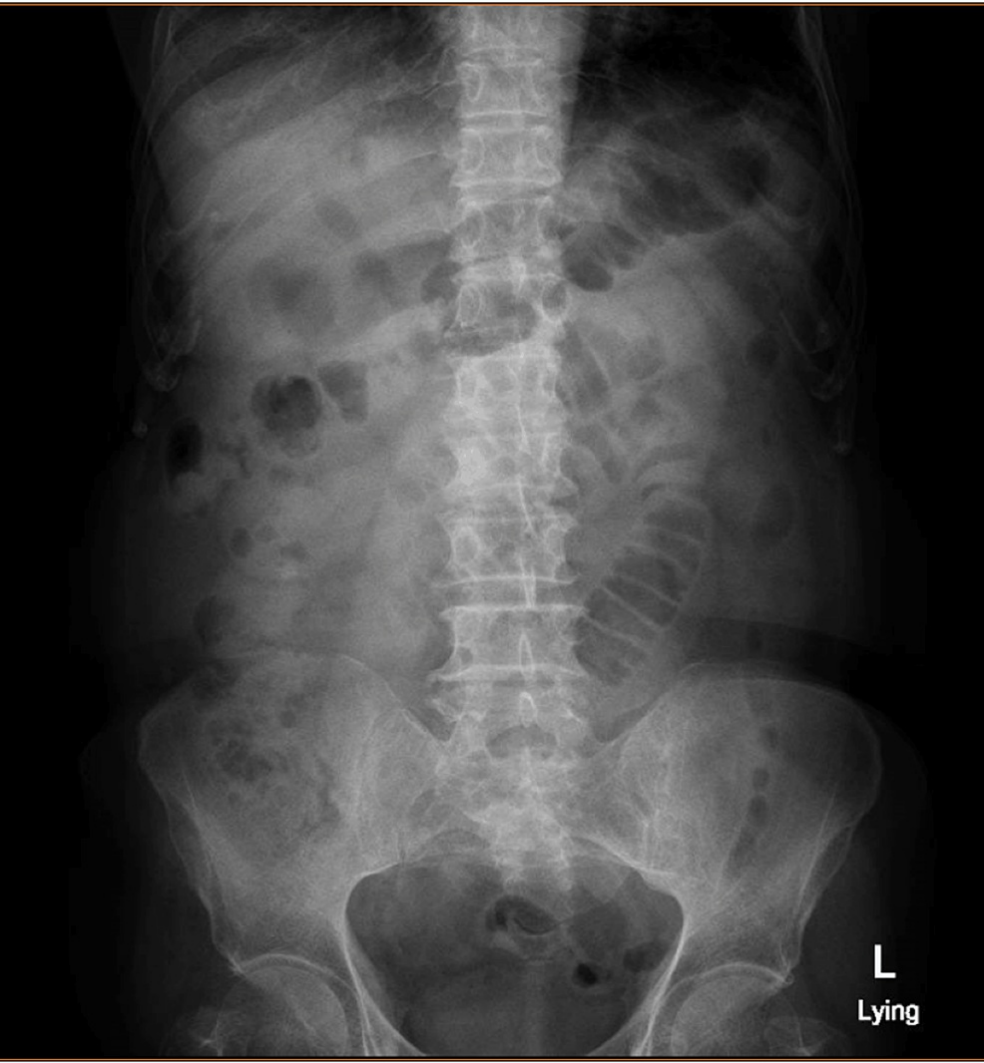
Preoperative abdominal X-ray showed dilated loops with excessive gases.

**Figure 2:**
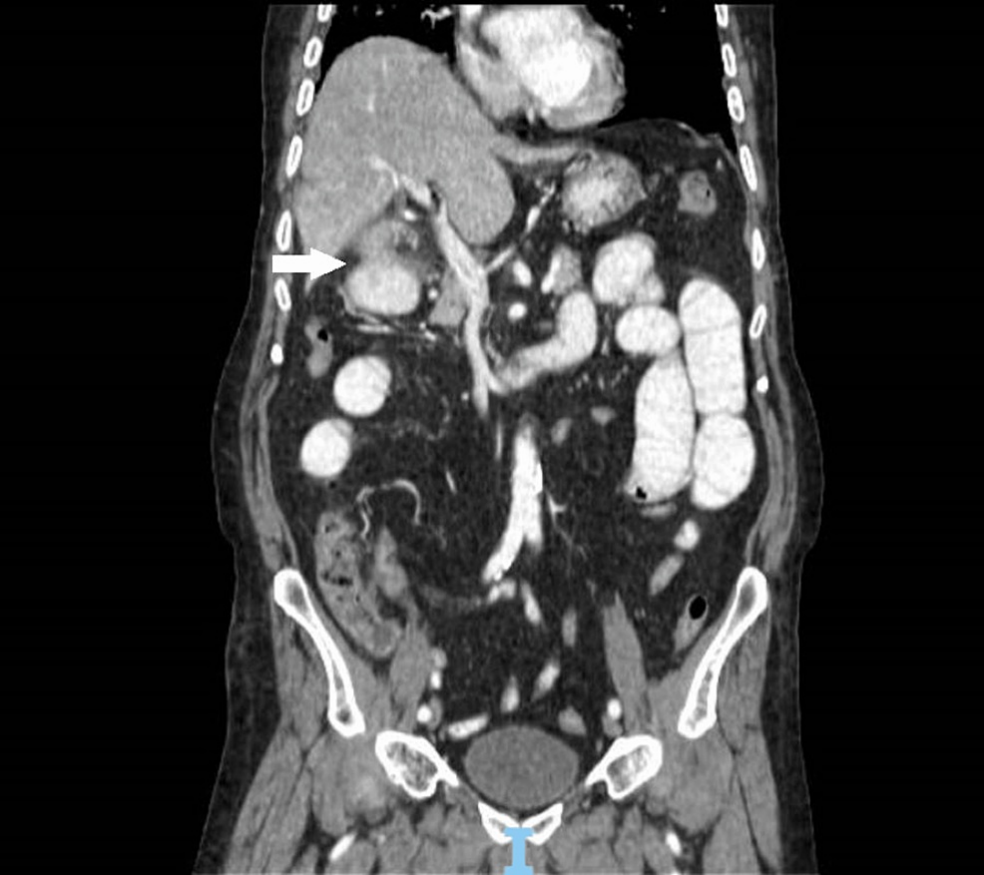
Fistula between the gallbladder and antrum The white arrow shows the connection between the gallbladder and gastric antrum.

**Figure 3:**
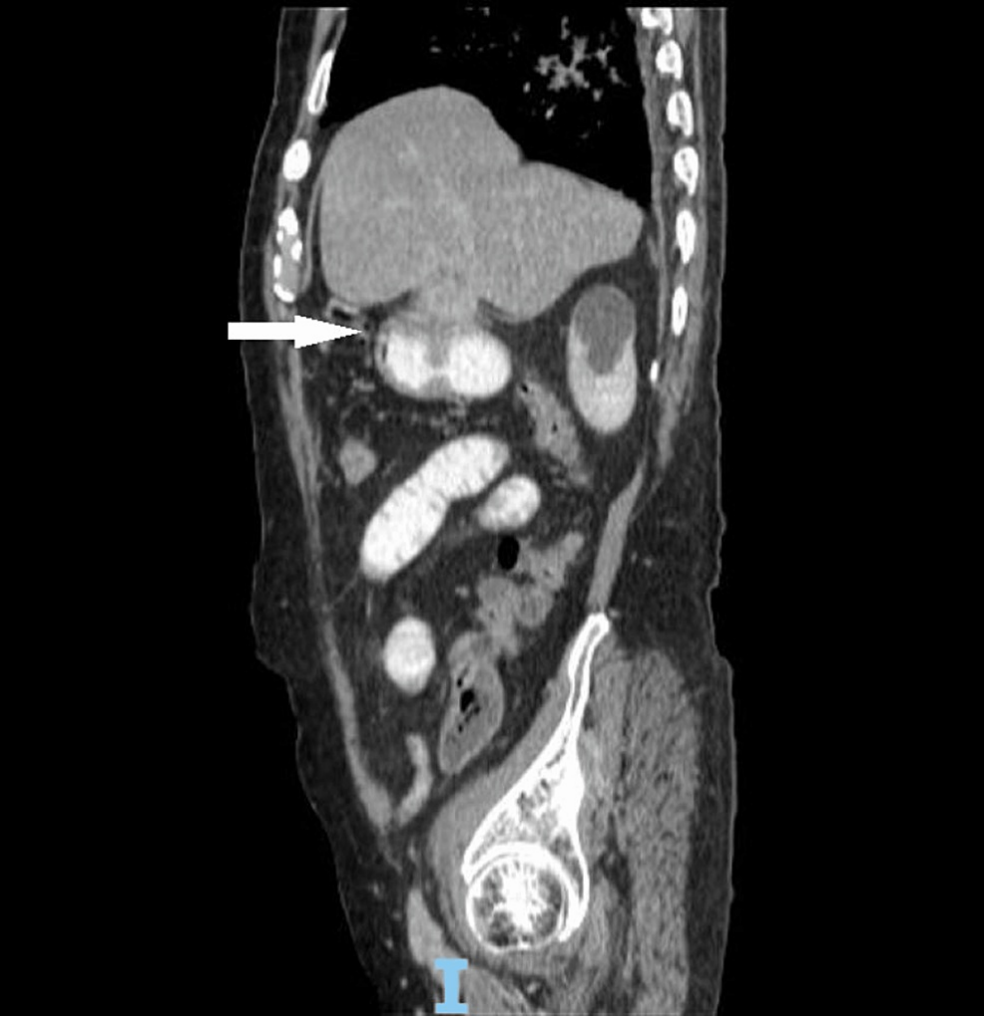
Sagittal view of the fistula between the gallbladder and the antrum The white arrow shows the connection between the gallbladder and the antrum.

**Figure 4:**
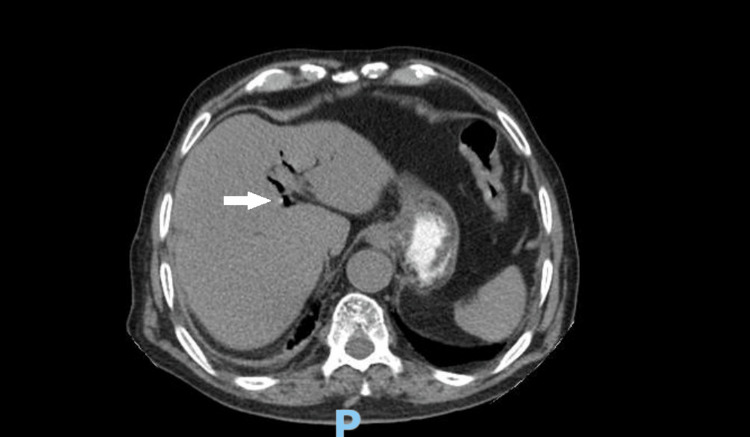
CT abdomen showing pneumobilia The arrow points to the air in the intrahepatic biliary tree.

**Figure 5:**
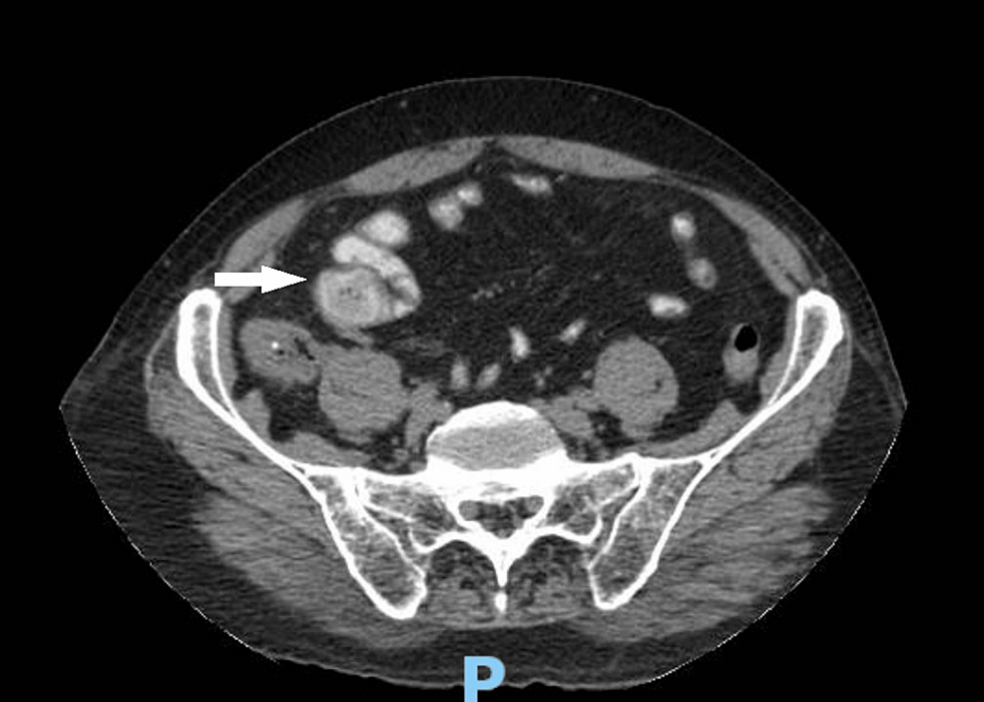
CT abdomen The arrow shows the Mercedes Benz sign with the proximal bowel loop dilated and collapsed distal loops.

A 98-year-old male presented to the emergency department of the Security Forces Hospital Program, Dammam with a five-day history of colicky periumbilical abdominal pain associated with non-bilious, non-blood-mixed vomiting and obstipation. He had no significant past medical or surgical history, and he was a nonsmoker. On examination, his vitals were as follows: blood pressure of 110/80, pulse rate of 86/minute, temperature of 37.1 Celsius, respiration rate of 18/minute, and SPO2 of 98% room air. He was not jaundiced and his abdomen was soft and lax with mild tenderness in the right hypochondrium on deep palpation. Routine blood tests were significant only for mild leukocytosis. The abdominal X-ray (AXR) showed dilated loops with excessive gases (Figure 1). Abdominal computed tomography (CT) with intravenous (IV) and oral contrast revealed dense adhesion between hepatic flexure and gallbladder (GB), absence of GBS with mild wall thickening suggesting chronic cholecystitis, suspected fistulous communication between gallbladder and the antrum (Figures 2-3), pneumobilia (Figure 4), SBO at distal jejunum with transitional zoon. This SBO was found to be secondary to impacted 18 mm single GBS which contains fissures filled with fluid and air which are arranged in a pattern resembling the Mercedes Benz sign (Figure 5), which presents in 50% of GBS cases. Proximal to the obstructing GBS, a moderately distended jejunal loop was located in the right lower abdomen, and distal to it were collapsed large intestinal loops, which contained no contrast inside their lumens. All the mentioned radiological findings suggest a gallstone ileus with a fistula between the inflamed gallbladder and antrum.

**Figure 6:**
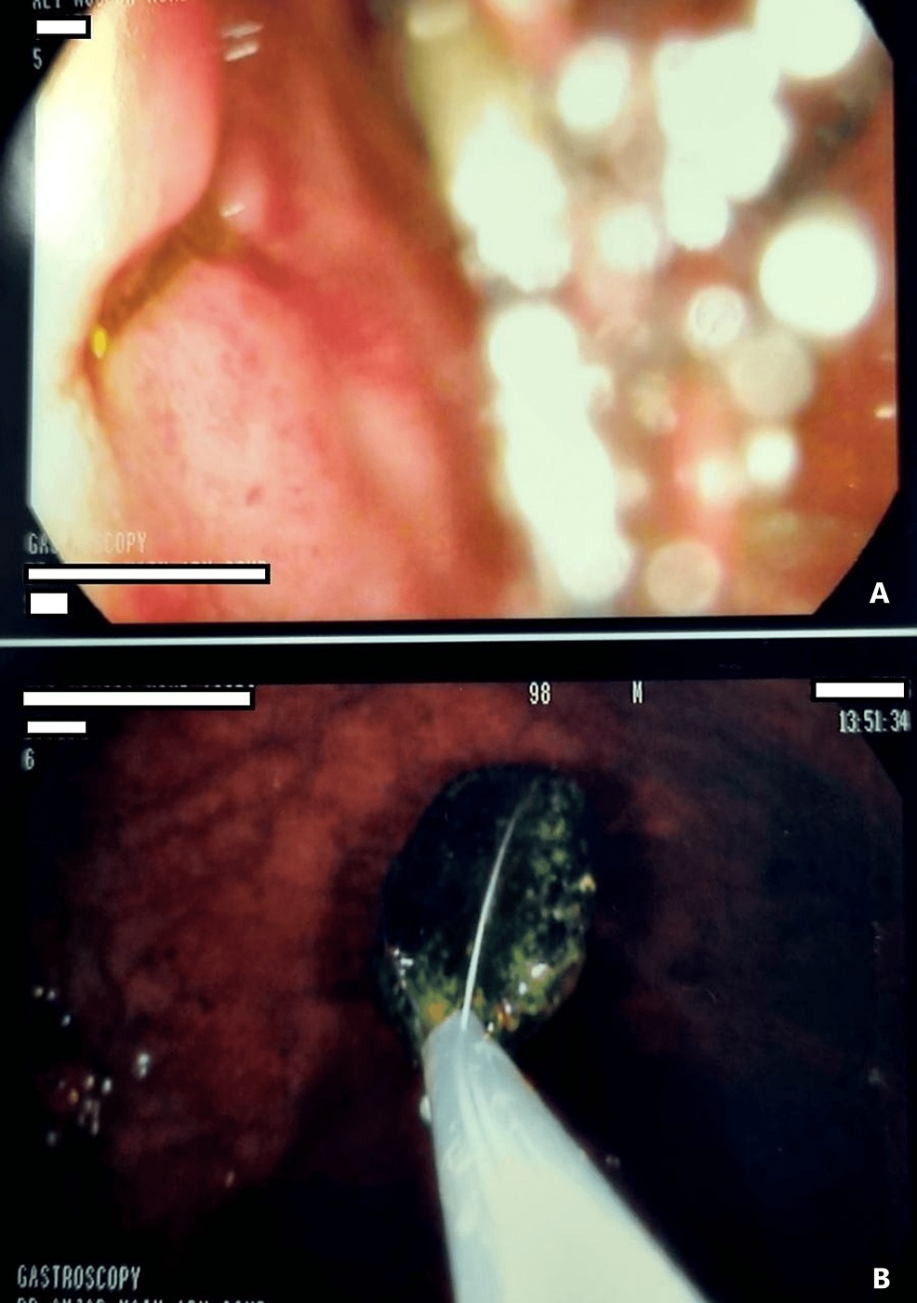
Upper endoscopy The upper panel (A) shows the bilious secretions and the lower panel (B) shows the gallbladder stone at the second part of the duodenum.

**Figure 7:**
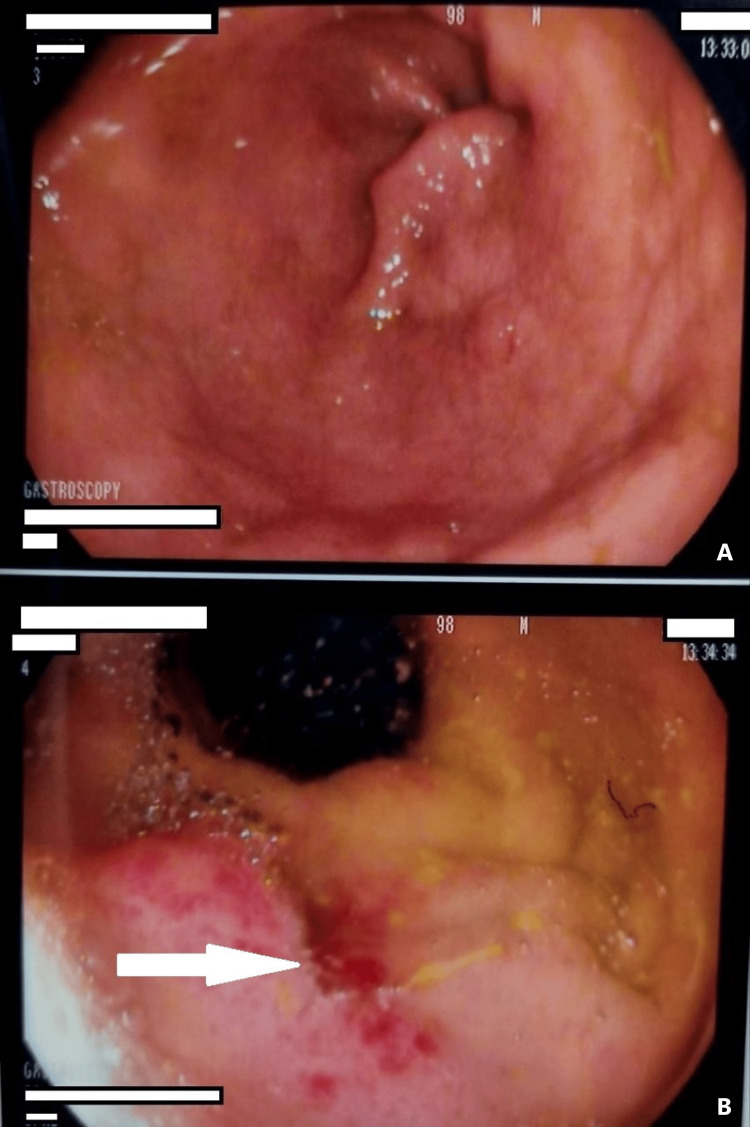
Upper endoscopic view The upper panel (A) shows normal gastric mucosa D2; in the lower panel (A) the arrow shows the ulcer at the first part of the duodenum.

An upper gastrointestinal endoscopy was done and showed competent cardia with bilious greenish secretions filling the stomach, normal gastric mucosa, and pylorus (Figure 6). The findings from the duodenum (D1) were the anterio-inferior wall ulcer of clean base 1 cm size (Figure 7), normal D2-D3, and the gallbladder stone retrieved from D2 by biopsy basket size of 1.5 cm.

**Figure 8:**
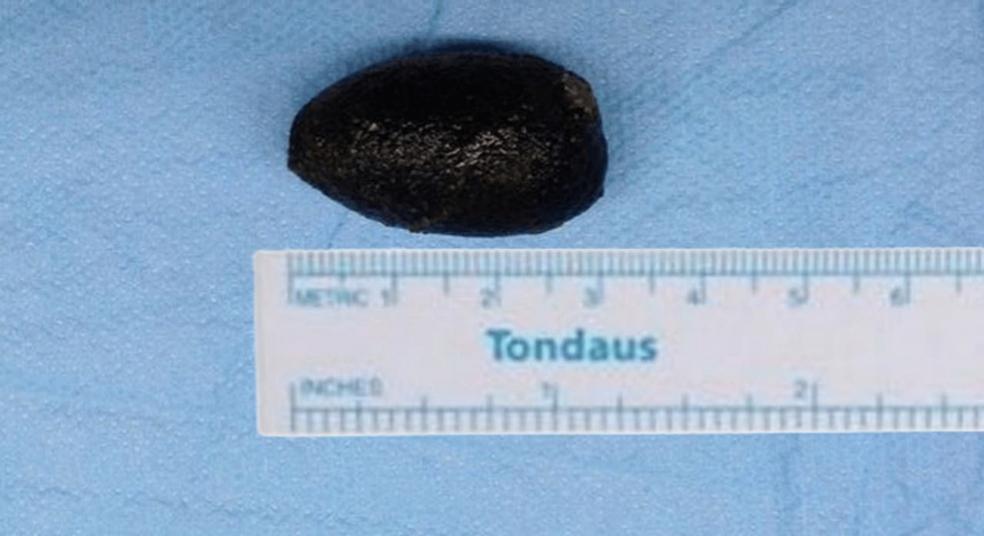
Gallstone retrieved from the jejunum

Our patient was taken to the operating room for an open exploratory laparotomy. Intraoperatively, a 3 cm GBS stone was found obstructing the distal jejunum (Figure 8). Our operative management consists of the removal of the obstructing stone through a longitudinal enterotomy then repaired transversely in a two-layer of non-absorbable suture. Due to extensive adhesions on the Calot’s triangle, we proceeded for partial cholecystectomy with a biopsy taken from the affected antrum and closing the cholecystic-antral fistula, then the antral repair was buttressed with an omental patch. Finally, the abdomen was closed, and one drain was placed in the subhepatic area. The patient had an uneventful postoperative course, and his drain output was decreased from 70 bloody ml/ day on the first postoperative day to 20 ml serous fluid on the third postoperative day, and the patient was discharged home on the same day. After two days, the patient returned to the emergency department complaining of pain and serosanguinous secretions from the lower part of the surgical site. On examination, the patient had mild tachycardia at 105 beats/min, a temperature of 38.4 Celsius, and local wound examination showed wound dehiscence in the lower part of the previous surgical wound. The patient was admitted to the surgical ward and then taken to the operation theatre for re-closure. The patient had a normal postoperative recovery, and he was discharged home on the third postoperative day. Over six months of regular clinic follow-up, our patient had no significant complaints. The histopathology report of the antral biopsy was negative for malignancy. 

## Discussion

CEF was first described by Courvoisier in 1890 [4]. It usually develops as a prolonged complication of cholelithiasis, but other factors lead to the formation of a fistula: gall bladder cancer, trauma, peptic ulcers, and diverticulitis [5,6]. According to Kaminsky's report, the CEF frequently occurs between the gallbladder and the duodenum (60%), due to their adjacency. then occurs with the colon, stomach, and common bile duct, (24%), (6%), and (5%) respectively.

In the literature, the CEF may present with a variety of symptoms: diarrhoea, abdominal pain, distention, jaundice, fever, sepsis, nausea, vomiting, SBO, steatorrhea, and weight loss [7]. The majority of cholecysto-antral fistulas usually affect female patients at age of 60 years [8]. The AXR and CT abdomen can reveal air in the biliary system, SBO, and ectopic gallstone in the right lower abdomen, while the ultrasound scan is of limited value in this case. Typically, the treatment is open surgery or laparoscopic exploratory laparotomy [9,10] for removal of the obstructing gallstone through a small enterotomy, followed by cholecystectomy and fistula closure if the patient is stable with clear anatomy; otherwise, it can be done later on, if indicated.

Both our study and literature share several key points: signs and symptoms of bowel obstruction, CT findings suggesting the diagnosis, and the surgical approach and management. On the other hand, we believe that our case is unique due to many aspects: firstly, to the best of our knowledge, this is the first reported and published case of ileus gallstone performed on a 98-year-old patient. Secondly, cases of biliary duodenal fistulae are not frequently discussed in the literature. Thirdly, the obstructing GS, in our case, presented the radiological Mercedes Benz sign which does not present in all cases of GBS. Fourthly, in our case, the migrating GBS was dislodged in the distal jejunum while typically, it is dislodged in the terminal ileum. Fifthly, our patient did not have previous biliary symptoms. Finally, we were able to remove another single GBS from the second part of the duodenum during the preoperative upper endoscopy. Therefore, the clinical suspicion of CEF and gallstone ileus should be present in mind in all adult patients who present with signs and symptoms of bowel obstruction even if there is no history of GBS. The priority in surgical management should be given to addressing the bowel obstruction with or without cholecystectomy and closure of the fistula according to the patient’s status and local anatomy. In the case of cholecysto-antral fistula, the preoperative upper endoscopy may be of diagnostic and therapeutic value.

## Conclusions

Cholecysto-antral fistula and gallstone ileus are infrequent but challenging complications of gallbladder stones and should be included in the differential diagnosis of bowel obstruction, especially in elderly patients with no history of gallbladder stones. The cornerstone of treatment is the proper diagnosis and timely surgical intervention to release the obstruction by enterotomy and extraction of the stones with or without cholecystectomy, and repair of the fistula according to the patient’s condition and surgical anatomy.
